# A Correlational Analysis of the Relationships among Intolerance of Uncertainty, Anxiety Sensitivity, Subjective Sleep Quality, and Insomnia Symptoms

**DOI:** 10.3390/ijerph16183253

**Published:** 2019-09-05

**Authors:** Marco Lauriola, R. Nicholas Carleton, Daniela Tempesta, Pierpaolo Calanna, Valentina Socci, Oriana Mosca, Federico Salfi, Luigi De Gennaro, Michele Ferrara

**Affiliations:** 1 Department of Social and Developmental Psychology, Sapienza, University of Rome, 00185 Rome, Italy; 2 Department of Psychology, University of Regina, Regina, SK S4S 0A2, Canada; 3 Department of Biotechnological and Applied Clinical Sciences, University of L’Aquila, 67100 L’Aquila, Italy (D.T.) (V.S.) (F.S.); 4 Department of Dynamic and Clinical Psychology, Sapienza, University of Rome, 00185 Rome, Italy; 5 Department of Psychology, Sapienza, University of Rome, 00185 Rome, Italy

**Keywords:** intolerance of uncertainty, anxiety sensitivity, sleep quality, insomnia severity, mediation analyses, depression, anxiety

## Abstract

In this study, we used structural equation modeling to investigate the interplay among Intolerance of Uncertainty (IU), Anxiety Sensitivity (AS), and sleep problems. Three hundred undergraduate students completed the Intolerance of Uncertainty Scale, the Intolerance of Uncertainty Inventory, the Anxiety Sensitivity Index, the Beck Depression Inventory, the State-Trait Anxiety Inventory, the Pittsburgh Sleep Quality Index and the Insomnia Severity Index. 68% and 40% of the students reported poor sleep quality or sub-threshold insomnia problems, respectively. Depression and anxiety levels were above the cut-off for about one-fourth of the participants. Structural equation modeling revealed that IU was strongly associated with AS, in turn influencing both insomnia severity and sleep quality via depression and anxiety. Significant indirect effects revealed that an anxious pathway was more strongly associated with insomnia severity, while a depression pathway was more relevant for worsening the quality of sleep. We discussed the results in the frameworks of cognitive models of insomnia. Viewing AS and IU as antecedents of sleep problems and assigning to AS a pivotal role, our study suggested indications for clinical interventions on a population at risk for sleep disorders.

## 1. Introduction

According to the American Psychiatric Association: Diagnostic and Statistical Manual of Mental Disorders [1], about one-third of the adult population are reported to have experienced one or more symptoms of insomnia during the past 6–12 months. Sleep problems, such as short sleep duration and poor sleep quality, are also widespread among university students [2,3,4,5,6]. Sleep problems have been shown to affect academic functioning and increase the risks of burnout, psychoactive substance use, fatigue, and comorbid physical or psychological problems [4,7,8,9].

Individuals with insomnia frequently have comorbid mental disorders, particularly depression or anxiety disorders [10,11]. Genetic and environmental factors contribute to both the onset of insomnia and variability in treatment [12]. Several authors have questioned whether symptoms of insomnia are an independent clinical predictor of affective disorders. Persistent insomnia may represent a risk factor, or an early symptom, of bipolar, depressive, and anxiety disorders [11]. A meta-analysis concluded that non-depressed people with insomnia have a twofold higher risk of developing depression than people with no sleep difficulties [13]. Correspondingly, depression and anxiety can be risk factors for insomnia. However, the nature of the relationship between depression and developing insomnia is controversial. For instance, two longitudinal studies showed that depression was a major risk factor for developing insomnia [14,15], while other studies based on retrospective reports suggested that prior depression was not associated with the onset of insomnia [16,17].

The role of anxiety has been less extensively studied than that of depression. Some researchers have suggested that anxiety may be a risk factor for the development of insomnia [14,15,16,17]. It has also been reported that insomnia often appears at about the same time or immediately after the onset of an anxiety disorder [17]. Similarly, a prospective study showed that high trait anxiety was associated with a threefold higher risk of developing insomnia [14]. 

Another one-year prospective study indicated that there was a bidirectional relationship between insomnia and both anxiety and depression [18]. The same study also suggested that the onset of anxiety or depression or the onset of insomnia could start a negative spiral in which one condition exacerbated the other. A systematic review also concluded that there was a bidirectional association between insomnia and anxiety and depression, suggesting that insomnia predicts and is predicted by anxiety and depression [19]. Regardless of the direction of causality, people with sleep problems often experience intrusive thoughts and uncontrollable worries during the pre-sleep period [20,21]. 

Previous research has overlooked the role of cognitive dispositions that might exacerbate worry and rumination, thus contributing to the continuation of sleep problems. Intolerance of uncertainty (IU) is one such disposition and encompasses the belief that uncertain events, however unlikely, are unfair, unacceptable, and threatening [22]. These beliefs make intolerance of uncertainty people more likely to experience overwhelming anxiety, which, in turn, may trigger enduring physiological arousal [23]. Levels of intolerance of uncertainty are higher in several affective disorder groups than in control groups, and variance in intolerance of uncertainty accounts for a substantial proportion of variance in symptoms severity independently of other critical constructs [24]. Hence, intolerance of uncertainty is considered a transdiagnostic factor for psychopathology, including generalized anxiety disorders and depression [25]. 

Anxiety sensitivity (AS) is another disposition that is thought to amplify anxiety responses. Anxiety sensitivity represents a fear of anxiety-related sensations, derived from the erroneous belief that these sensations might have harmful consequences [26]. The term anxiety sensitivity is used to refer to maladaptive responses to internal sensations and feelings of anxiety. Originally anxiety sensitivity was conceived as a unitary construct, but more recently it has been redefined as multifactorial, with a general factor and three group factors representing the somatic, psychological, and social domains [27,28].

Intolerance of uncertainty and anxiety sensitivity are separate constructs. Intolerance of uncertainty relates to the cognitive unacceptability of uncertainty and applies to a wide range of future events, whereas anxiety sensitivity is more tightly associated with uncertainty about, and misattributions of, specific internal states, such as fear of physical sensations, social situations, and intrusive thoughts [22]. This argument prompted Carleton and colleagues [22] to suggest that anxiety sensitivity is causally dependent on intolerance of uncertainty. Furthermore, there are reports of positive correlations between intolerance of uncertainty and anxiety sensitivity, but not so large as to suggest an overlap between the two constructs; each predicts a unique component of the variance in clinical outcomes [29,30]. 

The role of anxiety sensitivity and intolerance of uncertainty in sleep disorders has not been studied extensively, but there is evidence that anxiety sensitivity is associated with poor sleep quality in clinical samples, including individuals with panic disorders [31] and HIV-infected individuals, after controlling for negative affect and relevant medical covariates [32]. Following trait anxiety and depression, anxiety sensitivity was also the third-best predictor of insomnia severity in healthy adults [33]. The mental component of anxiety sensitivity seems particularly important as a predictor of poor sleep quality in university students [34]. To the best of our knowledge, research on intolerance of uncertainty and sleep quality includes only a recent study, showing that worry partially mediated the relationship between intolerance of uncertainty and poor sleep quality in an adolescent sample [35]. Although quantitatively scarce, the literature on cognitive dispositions is consistent with the cognitive models of sleep disturbances [20,21,36]. It maintains that rumination about negative events, excessive worry about future events, and cognitive intrusions, which are also frequent and disturbing amongst people high on intolerance of uncertainty and anxiety sensitivity, can hinder the onset of sleep in the pre-sleep period.

No single study has investigated the relationships among intolerance of uncertainty, anxiety sensitivity, subjective sleep quality, and insomnia symptoms. To fill this gap, we used structural equations (SEM) to model the correlations of cognitive dispositions and two subjective indicators of sleep quality and insomnia severity. In our models, we considered intolerance of uncertainty to be a stable, cognitive disposition reflecting a fundamental fear of the unknown and accounting for the severity and comorbidity of anxiety and depression symptoms [37,38,39]. Because intolerance of uncertainty is a key variable to understand worry and rumination, we assumed that intolerance of uncertainty was associated with poor sleep quality and more severe insomnia symptoms due to persistent rumination, worries, and intrusive thoughts [22,24]. Because anxiety sensitivity is associated with intolerance of uncertainty [29] and may also amplify the intensity of anxiety responses in the pre-sleep period [26,34], we expected anxiety sensitivity to play a pivotal role in the relationship between intolerance of uncertainty, poor sleep quality, and insomnia symptoms, also through anxiety and depression [32]. 

## 2. Materials and Methods 

### 2.1. Participants

The participants were 300 undergraduate students at the University of L’Aquila, Italy (57 men, 239 women, 4 of undisclosed gender). Their mean age was 21.38 years (SD = 4.02 years). An interviewer explained the study goals to potential participants and informed them that they could withdraw from the study, and their responses would have remained anonymous. We got verbal consent from all participants before data collection. The study was conducted in accordance with the Declaration of Helsinki. The ethical review board of the University of L’Aquila, Italy, approved the study (protocol n. 23038, 20 September 2016). The data were collected during the fall semester of the 2016–2017 academic year.

### 2.2. Measures

Intolerance of Uncertainty Scale (IUS-12): The IUS-12 is a 12-item scale describing negative beliefs about uncertainty and future events that might have negative consequences [40]. Participants gave responses using a five-point scale. The total score (α = 0.87, in this study) provides a valid measure of intolerance of uncertainty.

Intolerance of Uncertainty Inventory (IUI-A): The IUI-A is a 12-item scale developed to assesses core beliefs about intolerance of uncertainty [41]. The IUI-A items were devised to assess the tendency for a person to consider uncertainties in life to be unacceptable and threatening. These beliefs were added later to the theoretical definition of the intolerance of uncertainty construct and were not addressed in the IUS-12. The items were administered using a 5-point scale ranging from 1 (‘not at all characteristic of me’) to 5 (‘entirely characteristic of me’). The total score is a valid measure of intolerance of uncertainty as currently defined (α = 0.89, in this study).

Anxiety Sensitivity Index (ASI-3): The ASI-3 is an 18-item scale assessing the tendency to fear the symptoms of anxiety [28]. Responses were given using a five-point Likert scale. The ASI-3 yields three subscale scores: Fear of somatic sensations (α = 0.87), fear of loss of cognitive or psychological control (α = 0.83), and fear of publicly observable anxiety symptoms (α = 0.80).

The Beck Depression Inventory (BDI-II): The BDI-II [42] is a 21-item scale assessing affective, somatic, and cognitive symptoms of depression. Respondents rated the severity of each symptom using a 0 to 3 scale; higher scores represent more severe symptoms. The total score (α = 0.90 in this study) is a valid measure of depression severity. Ranges representing ‘light’ (14–19), ‘mild’ (20–29), ‘moderate’ (30–63) and ‘severe’ (64 or higher) symptoms of depression have been defined.

Trait anxiety: We used the 20-item trait subscale of the State-Trait Anxiety Inventory (STAI-Y) Italian version to assess the tendency to experience enduring anxiety states such as fear, worry, activation of the autonomic nervous system, and somatic symptoms [43]. Participants rated the habitual intensity of each symptom on a four-point Likert scale. The total score is a valid measure of trait anxiety (α = 0.90, in this study). The recommended clinical cut-off is >46 [44].

The Pittsburgh Sleep Quality Index (PSQI): The PSQI measures retrospective sleep quality and disturbances over one month [45]. It comprises nineteen questions that yield seven clinically derived component scores: Sleep quality, sleep latency, sleep duration, habitual sleep efficiency, sleep disturbances, use of sleeping medication, and daytime dysfunctions. All component scores range from 0 to 3, and the Global Score (α = 0.67 in the present study) ranges from 0 to 21. A conventional cutoff score of >5 is used to separate poor sleepers from good sleepers.

The Insomnia Severity Index (ISI): The ISI consists of seven items reflecting the criteria of the Diagnostic and Statistical Manual of mental disorders (DSM-IV) for an insomnia disorder [46]. These are sleep onset, sleep maintenance, early morning awakening problems, sleep dissatisfaction, the extent to which sleep problems interfere with daytime functioning, whether one’s sleep problems are noticeable to others, and the distress caused by sleep difficulties. Respondents rated symptom severity during the past two weeks using a five-point scale (0 = affected me very little to 4 = affected me a lot). The ISI yields a total score ranging from 0 to 28 (α = 0.82 in this study). The guidelines for interpretation are as follows: 0–7 = no clinically significant insomnia; 8–14 = subthreshold insomnia; 15–21 = clinical insomnia of moderate severity; 22–28 = severe clinical insomnia.

### 2.3. Data Analysis

**Figure 1:**
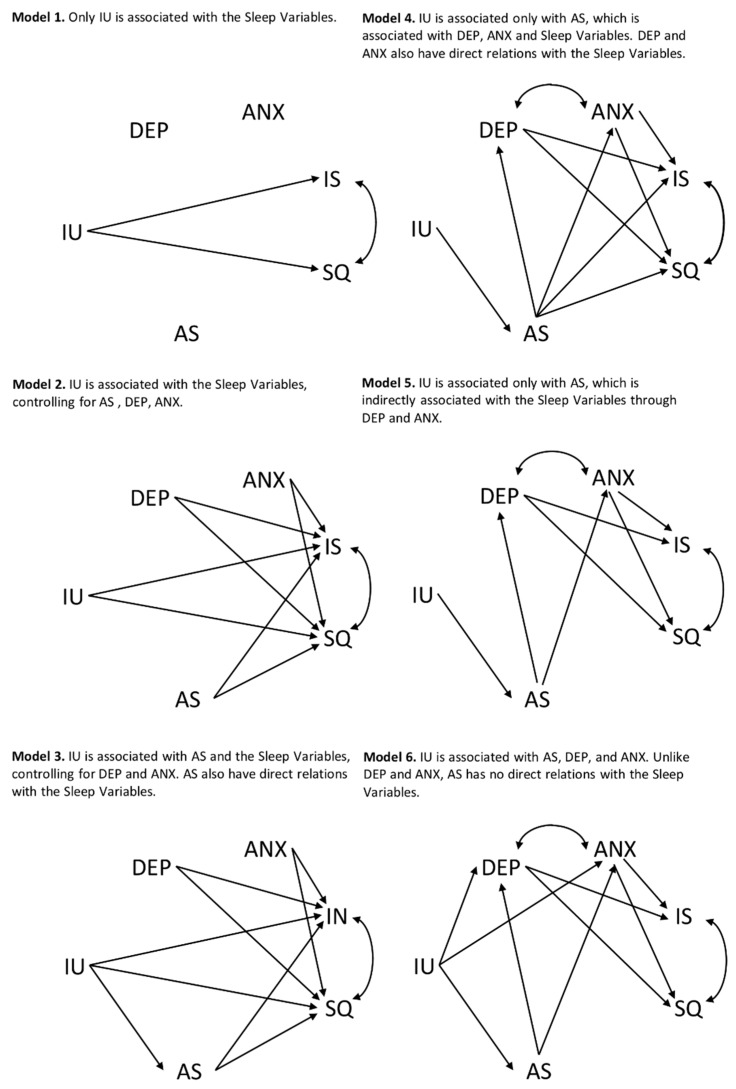
Schematic representation of six alternative structural equation models of sleep quality and insomnia severity. IU = Intolerance of Uncertainty; AS = Anxiety Sensitivity; DEP = Depression; ANX = Anxiety; IS = Insomnia Severity; SQ = Sleep Quality.

The study hypotheses were tested using a Structural Equation Modeling (SEM). Model 1 showed the hypothesis that only the intolerance of uncertainty was associated with the sleep variables (i.e., sleep quality and insomnia severity). Model 2 adjusted for the effects of anxiety sensitivity, anxiety, and depression. Model 3 added an indirect relationship of intolerance of uncertainty with the sleep variables through anxiety sensitivity, controlling for anxiety and depression. An indirect relationship is the product of the coefficients for the indirect paths that link two latent variables via other latent variables, and its significance can be tested using the approach outlined in Macho and Ledermann [47]. Model 4 displayed an indirect relationship of intolerance of uncertainty with anxiety, depression, and sleep variables through anxiety sensitivity, with anxiety and depression, which are also directly related to sleep variables. Model 5 was equivalent to Model 4, except that anxiety sensitivity was associated with anxiety and depression but not with the sleep variables. In Model 6, the relationship of intolerance of uncertainty with the sleep variables was through anxiety sensitivity, anxiety, and depression. The models are reported in Figure 1.

Each latent variable in the models was derived from related empirical indicators. For intolerance of uncertainty, we used the IUS-12 and the IUI-A scores. For anxiety sensitivity, we used the mental, cognitive, and social scores of the ASI-3. For anxiety, depression, sleep quality, and insomnia severity, we used item parcels, each of which was a composite score reflecting a set of homogeneous items. Because the proper use of parceling depends on the dimensionality of the items being combined [48], we ran preliminary principal component analyses to assess this. The parceling method produced unidimensional and convergent indicators for each latent variable supporting a proper use of the method (Table A1).

Because the data did not meet the multivariate normality assumption, the SEMs were estimated using the maximum likelihood robust (MLR) method using EQS 6.4, which corrects the parameters and fit indices of the models for non-normality [49]. Model fit was assessed using the following indices: Satorra–Bentler scaled χ^2^ (SBχ^2^), the robust version of comparative fit index (CFI), Bentler–Bonnett non-normed fit index (NNFI), and root-mean-square error of approximation (RMSEA), and the standardized root mean square residual (SRMR). The criteria for a good fit [50] are as follows: ≥0.95 for NNFI and CFI, ≤0.06 for RMSEA, and ≤0.08 for SRMR. 

Hierarchically nested models can be derived one from the other *only* adding or removing paths and can be compared to each other using the scaled Chi-square difference test [51]. If the test is not significant, the model with less free parameters should be preferred. Non-nested models can be compared descriptively, using the Akaike information criterion (AIC) and the AIC with the correction for finite sample sizes (CAIC); in both cases, the model with the lowest value offers the best fit to the data [52].

## 3. Results

### 3.1. Descriptive Analyses and Correlations

About two-thirds of the participants (*n* = 205, 68%) were poor sleepers, and 125 (40%) reported subthreshold insomnia. Twenty participants (7%) reported moderate or severe insomnia. About a quarter of participants (*n* = 76, 25%) reported above-threshold depression symptoms and a similar proportion (*n* = 78, 26%) reported above-threshold anxiety symptoms. As expected, above-threshold depression was strongly associated with insomnia severity (χ^2^ = 17.85; df = 1; *p* < 0.001), poor sleep quality (χ^2^ = 13.90; df = 1; *p* < 0.001), and anxiety (χ^2^ = 30.90; df = 1; *p* < 0.001). Anxiety was associated with insomnia severity (χ^2^ = 8.51; df = 1; *p* < 0.005), but not with poor sleep quality. These findings were confirmed by analyses of continuous data (see Table 1). Depression was moderately correlated with both sleep variables, whereas the effect sizes for the relationships between the sleep variables and anxiety were small-medium. The intolerance of uncertainty score was moderately correlated with scores on the anxiety sensitivity subscales, and both anxiety sensitivity and IU were associated with insomnia severity.

### 3.2. Structural Equations Modeling 

Both models 1 and 2 had a poor fit to the data (Table 2). Model 1 showed that intolerance of uncertainty was associated with insomnia severity (β = 0.25; *Z =* 3.38; *p* = 0.000) but not with sleep quality (β = 0.10; *Z =* 1.60; *p* = 0.109). Model 2 was an improvement on Model 1 (Δχ^2^ = 85.51; *df* = 6; *p* = 0.000). The set of independent variables in the model accounted for 35% and 31% of the variance in insomnia severity and sleep quality, respectively. Trait anxiety was the best predictor of insomnia severity (β = 0.50; *Z =* 7.59; *p* = 0.000), followed by depression (β = 0.23; *Z =* 3.65; *p* = 0.000). Likewise, trait anxiety (β = 0.39; *Z =* 6.44; *p* = 0.000) and depression (β = 0.37; *Z =* 6.17; *p* = 0.000) predicted poor sleep quality to a similar extent. Neither intolerance of uncertainty nor anxiety sensitivity were associated with the severity of insomnia symptoms, controlling for depression and anxiety. Anxiety sensitivity was also not related to sleep quality, while intolerance of uncertainty was unexpectedly associated with this variable in a negative direction (β = −0.25; *Z* = −3.60: *p* = 0.000). This negative suppression effect is a statistical artifact that arises in regression analyses when one of the independent variables (in this case intolerance of uncertainty) is more strongly associated with other independent variables (e.g., anxiety sensitivity, anxiety, and depression) than with the dependent variables in the model (e.g., sleep quality) [53]. 

Model 3 tested the indirect relationship of intolerance of uncertainty with the sleep variables through anxiety sensitivity while controlling for anxiety and depression. This model was an improvement over Model 2 (Δχ^2^ = 30.43; *df* = 1; *p* = 0.000) but failed to meet the acceptability criteria. Anxiety and depression were still the largest contributors to variance in the sleep variables. The path coefficients linking anxiety with insomnia severity and sleep quality were β = 0.50 (*Z* = 7.55; *p* = 0.000) and β = 0.38 (*Z* = 6.33; *p* = 0.000), respectively. The corresponding values for depression were β = 0.23 (*Z* = 3.72; *p* = 0.000) and β = 0.38 (*Z* = 6.35; *p* = 0.000). Although the path from intolerance of uncertainty to anxiety sensitivity was large (β = 0.56; *Z =* 6.50; *p* = 0.000), anxiety sensitivity was not associated with the sleep variables controlling for anxiety and depression, thus precluding the expected indirect relationship of intolerance of uncertainty with insomnia severity and sleep quality. As in Model 2, intolerance of uncertainty maintained a negative relationship with sleep quality (β = −0.26; *Z* = −3.03; *p* = 0.002). The variables in the equations accounted for 35% and 31% of the variance in insomnia severity and sleep quality, respectively.

Model 4 was an improvement on Model 3 (Δχ^2^ = 83.80; *df* = 1; *p* = 0.000) and was an acceptable fit, too (see Table 2). The path linking intolerance of uncertainty to anxiety sensitivity was large (β = 0.61; *Z =* 7.58; *p* = 0.000). In turn, anxiety sensitivity was linked with both anxiety (β = 0.72; *Z =* 6.49; *p* = 0.000) and depression (β = 0.67; *Z =* 6.19; *p* = 0.000). Trait anxiety was the only predictor of the severity of insomnia symptoms (β = 0.54; *Z =* 4.03: *p* = 0.000). Trait anxiety and depression predicted poor sleep quality: β = 0.36 (*Z* = 2.49; *p* = 0.013) and β = 0.40 (*Z* = 2.86; *p* = 0.004), respectively. Because intolerance of uncertainty was no longer specified to be related to the sleep variables, the suppression effect was detected for anxiety sensitivity with poor sleep quality (β = −0.22; *Z* = −2.10; *p* = 0.035). Anxiety sensitivity was not associated with insomnia severity. Using this model, the variables in the equations accounted for 37% and 33% of the variance in insomnia severity and sleep quality, respectively. Regarding the indirect relationships, anxiety sensitivity was associated with insomnia severity (Total indirect effect = 0.52; *Z =* 4.92; *p* = 0.000) and poor sleep quality (Total indirect effect = 0.52; *Z =* 5.04; *p* = 0.000) through anxiety, and depression. Likewise, intolerance of uncertainty was associated with insomnia severity (Total indirect effect = 0.23; *Z =* 3.42; *p* = 0.001) and poor sleep quality (Total indirect effect = 0.10; *Z =* 3.14; *p* = 0.002) through anxiety sensitivity, and then through anxiety and depression.

Model 5 was derived from Model 4, removing the path from anxiety sensitivity to the sleep variables to get rid of the negative suppression effects. Model 5 still was an acceptable fit to the data and was a non-significant loss of fit relative to Model 4 (Δχ^2^ = 4.03; *df* = 2; *p* = 0.133). Being more parsimonious, Model 5 accounted for 35% and 30% of the variance in insomnia severity and sleep quality, respectively. With few exceptions, the structural coefficients resembled those obtained in Model 4. The path linking intolerance of uncertainty to anxiety sensitivity was β = 0.60 (*Z =* 7.47; *p* = 0.000), and anxiety sensitivity was associated with anxiety (β = 0.71; *Z =* 6.52; *p* = 0.000) and depression (β = 0.66; *Z =* 6.21: *p* = 0.000). Trait anxiety was again the only predictor of insomnia severity (β = 0.45; *Z =* 3.94; *p* = 0.000). Different from Model 4, only depression was associated with poor sleep quality (β = 0.35; *Z* = 2.71; *p* = 0.007). The indirect relationships were consistent with those estimated in the previous analysis. Anxiety sensitivity was associated with insomnia severity (Total indirect effect = 0.43; *Z =* 5.93: *p* = 0.000) and poor sleep quality (Total indirect effect = 0.39; *Z =* 6.04: *p* = 0.000) through anxiety, and depression. Intolerance of uncertainty was associated with insomnia severity (Total indirect effect = 0.26; *Z =* 3.99: *p* = 0.000) and poor sleep quality (Total indirect effect = 0.24; *Z =* 4.05: *p* = 0.000) through anxiety sensitivity, and then through anxiety, and depression. 

Model 6 was obtained from Model 5 by adding paths from intolerance of uncertainty to both anxiety and depression. Model 6 still accounted for 35% and 30% of the variance in insomnia severity and sleep quality, respectively, however outperformed Model 5 in terms of absolute (Table 2) and relative fit (Δχ^2^ = 11.83; *df* = 2; *p* = 0.002). 

**Figure 2:**
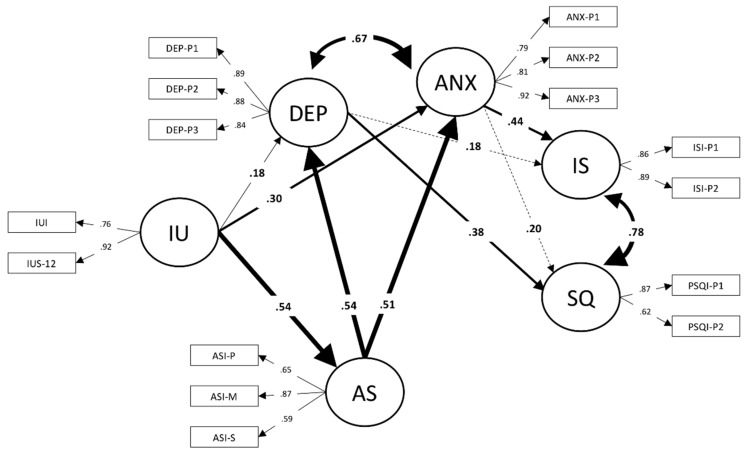
Structural equation model of insomnia severity and sleep quality. IU = Intolerance of Uncertainty; AS = Anxiety Sensitivity; DEP = Depression; ANX = Anxiety; IS = Insomnia Severity; SQ = Sleep Quality. The solid lines represent statistically significant paths (*p* < 0.05). The dashed lines represent not significant paths. The thickness of the lines representing structural relationships is proportional to the effect size of the path coefficient.

The model’s parameter estimates are reported in Figure 2. All the factor loadings were statistically significant, and in most cases, the coefficients were very large. No negative suppression effects were detected. The structural coefficients showed that intolerance of uncertainty was more strongly associated with trait anxiety than with depression. The largest effect size for intolerance of uncertainty was with anxiety sensitivity, which in turn was associated with trait anxiety and depression. As shown in Model 5, trait anxiety was associated with insomnia severity: While depression was associated with poor sleep quality. 

The total indirect effect of intolerance of uncertainty with insomnia severity and sleep quality were 0.33 (*Z =* 4.47; *p* = 0.000) and 0.29 (*Z =* 3.86; *p* = 0.000), respectively. However, the relative effect sizes of the paths depicted in Figure 2 suggested two possible indirect relationships for intolerance of uncertainty with the sleep variables. On the one hand, intolerance of uncertainty might be indirectly linked with insomnia severity through an increase in anxiety sensitivity, and anxiety symptoms combined with anxiety sensitivity. On the other hand, intolerance of uncertainty might be linked with sleep quality through an increase in anxiety sensitivity, and depression symptoms associated with anxiety sensitivity. To assess the statistical significance of these relations, we broke down the total indirect effects into specific components [47].

Regarding insomnia severity, the indirect effect of intolerance of uncertainty through anxiety sensitivity, and subsequently through anxiety was significant (Specific indirect effect = 0.13; *Z =* 2.58; *p* = 0.010), while that through depression failed to attain statistical significance (Specific indirect effect = 0.06; *Z =* 1.43; *p* = 0.153). The corresponding tests for sleep quality showed that the indirect effect of intolerance of uncertainty through anxiety sensitivity, and subsequently through anxiety was not significant (Specific indirect effect = 0.06; *Z =* 1.39; *p* = 0.010), while that through depression attained the conventional levels (Specific indirect effect = 0.06; *Z =* 2.36; *p* = 0.164). 

## 4. Discussion

Cognitive theories of psychopathology maintain that intolerance of uncertainty and anxiety sensitivity confer a higher risk of developing anxiety and depression [39]. Sleep research has also shown that, along with anxiety and depression, anxiety sensitivity can uniquely predict poor sleep quality in clinical samples [31,32] and insomnia symptoms in healthy adults [33], while intolerance of uncertainty has been less studied with sleep problems [35]. This literature has inspired our research and provided a rationale for examining the relations of intolerance of uncertainty and anxiety sensitivity with the intensity of insomnia symptoms and the quality of sleep, controlling for depression and anxiety, in a population putatively at high risk of developing sleep disturbances [3,5,54].

Our study showed that intolerance of uncertainty and anxiety sensitivity are involved in the process that links personality to sleep problems. Both were associated with two established subjective indicators that we have used to profile the severity and the quality of sleep disturbances [55]. Different from previous research carried out on adolescents [35], we found an effect size smaller than expected for the correlation between intolerance of uncertainty and sleep quality. In the absence of a better explanation, we are inclined to attribute this unexpected finding to the different population used in the present study. For instance, research has shown that young adult students and workers have different beliefs and different ways of managing their uptime [56]. Similarly, young adults might differ from adolescents as it regards sleep regulation processes, which might affect the relationship between intolerance of uncertainty and sleep quality.

Another unexpected finding was that neither anxiety sensitivity nor intolerance of uncertainty accounted for substantial proportions of variance in the sleep variables when controlling for anxiety and depression, while a previous study suggested that anxiety sensitivity was to provide a unique contribution in multivariate analyses [34]. In addition to the sample-specific factors mentioned above as an account for contrasting results, it is worth noting that the previous research controlled for trait neuroticism, that is a broad personality trait correlated with—but not overlapping to—anxiety and depression symptoms [34]. Our research instead controlled for specific tendencies to experience enduring anxiety and depression states, using established symptoms scales, which could be more strongly associated with poor sleep quality, insomnia severity, and anxiety sensitivity, than a personality characteristic like neuroticism.

At first glance, these findings suggested the role of cognitive dispositions to be less than was supposed. However, they also prompted a new perspective, in which the associations of intolerance of uncertainty with the sleep variables might be best represented by indirect relationships like a significant increase in anxiety sensitivity and psychological symptoms combined with anxiety sensitivity. Indeed, using structural equations modeling, we showed that intolerance of uncertainty and anxiety sensitivity were differently associated with insomnia severity and poor sleep quality. Anxiety sensitivity was more closely associated with psychological symptoms of anxiety and depression, and to the sleep variables than intolerance of uncertainty. This latter disposition was more proximal to psychological symptoms. About one-third of the variance in insomnia severity and poor sleep quality was accounted for by the indirect relationships mentioned above and well represented in Model 6, which had an excellent fit to the data. 

In keeping with previous research, anxiety sensitivity had a central role [31,32,33]. Our findings are consistent with the view that exaggerated concerns about the real or presumed consequences of anxiety might affect the sleep variables through the magnification of contingent anxiety and depression states. This interpretation is consistent with the trait-characteristics of anxiety sensitivity that not only amplifies anxiety-related sensations but also to spread to the cognitive and somatic symptoms of depression [39,57]. Specifically, it looks like the anxiety sensitivity mental component is most strongly related to depression than the physical and social components which affect anxiety symptoms [57,58]. One previous study has highlighted that the mental component was the facet of anxiety sensitivity that best accounted for poor sleep quality in university students [34]. In keeping with this literature, our correlation table showed that the anxiety sensitivity mental score was associated with depression, anxiety, insomnia severity and sleep quality to a greater extent than other anxiety sensitivity components (Table 1). Incidentally, the anxiety sensitivity mental score was also the best marker of the latent variable representing anxiety sensitivity in Model 6, again supporting the centrality of cognitive concerns for university students reporting sleep problems (Figure 2). 

Previous research has shown that intolerance of uncertainty is a cognitive vulnerability factor for enduring and intense worry, a maladaptive process underlying anxiety disorders and sleep problems in adolescents [35]. The significant paths represented in Model 6 are consistent with this view, but our data also suggested that the greater dispositional fear of the unknown might be associated with a greater apprehension of anxiety-related sensations, which may increase the risk of developing or maintaining sleep problems as said above. Different from previous studies, our analyses allowed us to compare the relative strength of specific indirect effects. Thus, Model 6 revealed an anxiety pathway through which intolerance of uncertainty might have a more negative impact on the risk of insomnia than on sleep quality. This interpretation is consistent with cognitive-behavioral models of insomnia [20,36], which have heuristic value in describing the psychological mechanisms involved in insomnia and their interrelationships with one another [18]. Accordingly, intolerance of uncertainty might increase the likelihood that a person will engage in cognitive activities (e.g., worrying) capable of heightening cognitive arousal and triggering autonomic arousal reactions (e.g., hypervigilance, palpitations) in the pre-sleep period. In turn, anxiety sensitivity might amplify those reactions escalating their potential for inhibiting normal sleep patterns [59]. 

The specific indirect effects in our Model 6 also suggested that there might be a depression pathway through which intolerance of uncertainty might have a more negative effect on sleep quality than on insomnia severity. Poor sleep quality is a typical characteristic of depression, with a likely bidirectional relationship [18,60]. Poor sleepers often complain about intrusive and uncontrollable ruminative thinking during the pre-sleep period and report using ineffective mental control strategies to try to suppress unpleasant thoughts [20]. Our results are consistent with these considerations and add to the finding that ruminative thinking and irrational thoughts are a depression-specific form of anxiety sensitivity, with cognitive symptoms easily misinterpreted as signs of impending insanity [58,61]. Both the fear of loss of cognitive control and the experience of such loss of control can induce demoralization, hopelessness, and despair, which might contribute to both depression and poor sleep quality [21].

Insomnia severity and poor sleep quality were intertwined. However, Model 6 revealed different indirect relationships for intolerance of uncertainty and anxiety sensitivity with the two sleep variables. In the above paragraphs, we have discussed these findings from a personality perspective. However, it is worth pointing out that the subjective indicators of sleep that we have used in our study are not entirely interchangeable and may reflect conceptual and empirical distinctions in the assessment of sleep disturbances. Except for fatigue, a hallmark symptom of insomnia, the Pittsburgh Sleep Quality Index has the broadest coverage of sleep problems [55]. By contrast, the Insomnia Severity Index is limited to DSM-IV criteria for an insomnia disorder and assesses symptom severity rather than frequency and quality of sleep disturbances. For instance, it does not include symptoms listed in the PSQI, such as poor sleep due to pain, urinary frequency, breathing difficulty, snoring, and so forth, which are relevant to other sleep disturbances. In light of these facts, we cautiously speculate that the anxious pathway might link intolerance of uncertainty and anxiety sensitivity to the risk of developing or maintaining severe insomnia symptoms. By contrast, the depressive pathway might not necessarily link intolerance of uncertainty and anxiety sensitivity to insomnia but rather to poor sleep quality in general.

Despite methodological strengths, such as testing theoretical claims in non-experimental data, and simultaneously addressing complex inter-relationships between variables, our study is not exempt from limitations. First, our findings are entirely based on cross-sectional data. We cannot rule out alternative explanations regarding the direction of the statistical associations reported in this paper. For instance, insomnia severity and poor sleep quality might precede the onset insurgence of depression or anxiety symptoms; it is still entirely possible that impaired sleep reinforces dysfunctional cognitive dispositions associated with psychological symptoms. Because causal inferences cannot properly be made without active control over the variables concerned, randomized trials or natural experiments, proving the existence of causal relations, is a challenge for future research. 

Second, our study was based on a convenience sample. University students, especially psychology major, are not representative of the Italian population, at least in terms of age, gender, education, and personality characteristics [62]. For instance, the prevalent female gender in our sample might have heightened the average anxiety and depression ratings. Moreover, because university students have irregular sleep patterns, about one-third of the students were poor sleepers, and forty percent reported sub-threshold insomnia. So, although we admit that there might be a potential selection bias in our study, the sample characteristics increased the salience of our findings for a population at risk for sleep disturbances. Unfortunately, however, our study did not directly assess the many factors that could influence sleep patterns in this population (e.g., academic stress, which is very common in students). Therefore, future research should also examine whether our conclusions are robust to other potentially confounding factors not considered in the present analyses.

A third limitation was that the effect sizes for key correlations, path coefficients, and indirect effects were only small–medium (according to Cohen’s standards). However, the total indirect effects estimated in Model 6 were large enough to support the claim that intolerance of uncertainty and anxiety sensitivity contributed significantly to sleep problems. For instance, based on the standardized indirect effects, we appraised increases in insomnia severity and poor sleep quality due to intolerance of uncertainty as 0.33 and 0.29 standard deviations, respectively.

Its limitations notwithstanding, our study is the first to model the interplay of intolerance of uncertainty and anxiety sensitivity in the context of insomnia symptoms and sleep quality. Our models also have implications for clinical practice. Intolerance of uncertainty and anxiety sensitivity are both therapeutically malleable constructs, namely psychological variables that can be reduced through targeted interventions. Dysfunctional beliefs about physical and cognitive consequences of anxiety might be successfully treated. Likewise, maladaptive responses to uncertainty, such as thought suppression, could be modified [63,64]. For instance, intervention studies could also be used to determine whether reducing intolerance of uncertainty or anxiety sensitivity of patients suffering insomnia leads to concurrent improvements in the severity of psychological and sleep symptoms, as described by our statistical model. Although we hope that the results obtained will be useful for clinical interventions on sleep disorders, our wish is limited by having worked only on healthy subjects. Thus, an avenue for future research might be manipulating intolerance of uncertainty and anxiety sensitivity to produce a beneficial effect on sleep in putatively high-risk populations.

## 5. Conclusions

In conclusion, this study suggested that intolerance of uncertainty and anxiety sensitivity were both involved in the process that links personality to sleep problems. More specifically, we showed that intolerance of uncertainty was strongly associated with anxiety sensitivity, in turn influencing both insomnia severity and sleep quality via depression and anxiety. Significant indirect effects suggested an anxious pathway to be more strongly associated with insomnia severity, while a depression pathway appeared more relevant for worsening the quality of sleep. Viewing anxiety sensitivity and intolerance of uncertainty as antecedents of sleep problems and assigning to anxiety sensitivity a pivotal role, our study suggested indications for clinical interventions on populations at risk for sleep disorders.

## Figures and Tables

**Table 1:** Descriptive Statistics and relationships between variables.

Variables	M	SD	1.	2.	3.	4.	5.	6.	7.	8.
1. Intolerance of Uncertainty (IUS-12)	27.70	8.63	1							
2. Intolerance of Uncertainty (IUI-A)	25.90	8.08	0.70 ***	1						
3. Anxiety Sensitivity Physical (ASI-3)	4.06	4.61	0.27 ***	0.30 ***	1					
4. Anxiety Sensitivity Social (ASI-3)	6.87	4.84	0.33 ***	0.31 ***	0.43 ***	1				
5. Anxiety Sensitivity Mental (ASI-3)	4.26	4.22	0.43 ***	0.35 ***	0.57 ***	0.50 ***	1			
6. Depression (BDI-II)	9.86	8.30	0.41 ***	0.35 ***	0.38 ***	0.34 ***	0.53 ***	1		
7. Trait Anxiety (STAI-Y)	40.12	9.29	0.50 ***	0.40 ***	0.40 ***	0.37 ***	0.57 ***	0.73 ***	1	
8. Sleep Quality (PSQI)	6.00	2.74	0.06	0.03	0.18 **	0.11	0.21 ***	0.42 ***	0.41 ***	1
9. Insomnia Severity (ISI)	7.14	4.51	0.22 ***	0.19 ***	0.24 ***	0.15 ***	0.33 ***	0.48 ***	0.51 ***	0.67 ***

N = 300; *** *p* < 0.001, ** *p* < 0.01, * *p* < 0.05.

**Table 2:** Summary Table of Fit Statistics for Structural Equation Models.

Model	ML χ^2^	DF	MLR χ^2^	NNFI *	CFI *	SRMR	RMSEA *	95% CI	AIC *	CAIC *
1	714.56	87	612.88	0.638	0.697	0.323	0.142	0.131–0.153	438.9	29.7
2	601.35	81	521.00	0.672	0.747	0.290	0.135	0.124–0.146	359.0	−22.0
3	534.56	80	468.41	0.707	0.777	0.283	0.127	0.116–0.138	308.4	−67.9
4	164.84	79	149.88	0.946	0.959	0.050	0.055	0.041–0.068	−8.1	−379.7
5	169.29	81	153.91	0.946	0.958	0.056	0.055	0.041–0.068	−8.1	−389.1
6	150.16	79	138.17	0.955	0.966	0.050	0.050	0.036–0.063	−19.8	−391.4

The χ^2^ values were significant for all models; NNFI * = robust version of the non-normed fit index; CFI * = robust version of the comparative fit index; RMSEA * = robust version of the Root Mean Square Error of Approximation; 95% CI = 95% confidence interval of the RMSEA; AIC * = Robust version of the Akaike Information Criterion; CAIC * = Robust version of the Consistent Akaike Information Criterion.

## Appendix A

**Table A1:** Assessment of unidimensionality and convergence of the parcels used to measure the latent variables in structural equation modeling.

Domain	Parcel	Items	Parcel Mean	Parcel SD	PCA Eigenvalues	Items in the Parcel	Cronbach’s Alpha	Parcel-Domain Correlation *
BDI	1	1, 4, 7, 10, 13, 16, 19	0.52	0.46	**2.90**; 0.86; 0.80; 0.75; 0.61; 0.59; 0.51	7	0.75	0.80
	2	2, 5, 8, 11, 14, 17, 20	0.49	0.46	**3.08**; 0.86; 0.75; 0.68; 0.63; 0.54; 0.45	7	0.79	0.82
	3	3, 6, 9, 12, 15, 18, 21	0.40	0.38	**2.67**; **1.03**; 0.85; 0.77; 0.63; 0.56; 0.49	7	0.72	0.80
STAI	1	1R, 10R, 19R, 2, 5, 11, 15	2.03	0.49	**2.85**; 0.99; 0.92; 0.78; 0.71; 0.48; 0.23	7	0.73	0.79
	2	7R, 13R, 3, 8, 12, 17, 20	2.16	0.60	**3.23**; 0.91; 0.89; 0.62; 0.49; 0.44; 0.43	7	0.82	0.78
	3	6R, 16R, 4, 9, 14, 18	2.22	0.55	**2.41**; **1.03**; 0.92; 0.60; 0.56; 0.48	6	0.69	0.83
PSQI	1	1, 3, 5, 7	1.01	0.70	**1.71**; 0.91; 0.81; 0.57	4	0.55	0.76
	2	2, 4, 6	1.06	0.68	**1.39**, 0.84, 0.76	3	0.40	0.76
ISI	1	1, 3, 5, 7	0.94	0.41	**1.11**; 0.82; 0.68; 0.38	4	0.68	0.47
	2	2, 4, 6	0.74	0.50	**1.77**; 0.77; 0.46	3	0.65	0.47

* Corrected for overlap. R = Reverse scored item. Bold type fonts indicate eigenvalues-greater-than-one in principal component analysis.
